# Is procrastination among students lower in group work? Evidence from a registered field experiment

**DOI:** 10.1111/bjep.70069

**Published:** 2026-03-10

**Authors:** Markus Koppenborg, Joachim Hüffmeier, Katrin B. Klingsieck

**Affiliations:** ^1^ Q^3^ – Evaluation, Development & Accreditation University of Cologne Cologne Germany; ^2^ Department of Social, Work, and Organizational Psychology TU Dortmund University Dortmund Germany; ^3^ Department of Sport Science and Physical Education University of Agder Kristiansand Norway; ^4^ Department of Psychology Paderborn University Paderborn Germany

**Keywords:** contextual prevention, expectancy‐value theory, group work, task structure

## Abstract

**Background:**

Research on procrastination mostly focuses on person‐related antecedents and neglects situational and social factors, such as group work. Prior research indicates that conjunctive and additive group work may increase individual effort and performance as compared to individual work.

**Aims:**

Based on these findings, we investigate whether conjunctive and additive group work may also help reduce procrastination as compared to individual work.

**Methods:**

In a registered field experiment, *N* = 218 students with high levels of trait procrastination worked on an academic task over the course of 10 days in one of three conditions (individual work vs. conjunctive group work vs. additive group work). Dependent variables comprised task procrastination, task performance, and positive and negative task‐related affect.

**Results:**

Regarding conjunctive group work, results are mixed, with some evidence that conjunctive group work leads to lower procrastination as compared to individual work. Both types of group work resulted in higher negative task‐related affect when assessed prospectively. No other effects were found.

**Conclusions:**

The findings contribute to the idea that targeted changes in the learning environment, such as the implementation of group work, may help reduce procrastination.

## INTRODUCTION

Procrastination, putting off against better judgement, is commonly known as a self‐regulation failure (e.g., Steel, 2007). Previous procrastination research and interventions have focused on person‐related antecedents of procrastination. A growing body of literature, however, suggests that situational and social factors may also influence procrastination episodes (e.g., Bäulke & Dresel, 2023; Svartdal et al., 2020). One social factor, which might have an effect on procrastination and which is very common in higher education, is group work among students. Group as compared to individual work can increase individual effort and performance, if the own contribution is perceived as indispensable for the group product (Johnson & Johnson, 2002; Torka et al., 2021; Weber & Hertel, 2007). This typically occurs in conjunctive group work, in which the group performance is determined by the weakest group member (Steiner, 1972). Here, the least capable group member exhibits effort and performance gains (Torka et al., 2021; Weber & Hertel, 2007). This might also apply, albeit to a lesser extent, to additive group work, in which the group performance is determined by all members' contributions (Steiner, 1972). Here, the weakest group member's contribution can be compensated by stronger group members, rendering perceptions of indispensability less pronounced.

This raises the question whether conjunctive and additive group work can also reduce procrastination. There is initial pertinent evidence from a qualitative study (Klingsieck et al., 2013) and a vignette study (Koppenborg et al., 2024), whose generalizability to real settings is limited. In this registered field experiment,^1^ we investigated the effects of conjunctive and additive group work versus individual work on procrastination, monitoring positive and negative affect as key variables in procrastination research and exploring indispensability perceptions as a potential underlying variable. The results should be relevant not only for interventions against procrastination but also for a new approach that seeks to prevent it by changing the learning environment.

## LITERATURE REVIEW AND HYPOTHESES

### Procrastination, interventions and the higher education environment

Procrastination has been defined as “the voluntary delay of an intended course of action despite expecting to be worse of for the delay” (Steel, 2007, p. 66) and has been coined as the “quintessential self‐regulatory failure” (Steel, 2007, p. 65). As an umbrella term, self‐regulation includes the regulation of cognition, emotion and behaviour for the sake of reaching a personal goal (Inzlicht et al., 2021). In the cognitive dimension, procrastination has been associated, with deficits in executive functions (e.g., Gustavson et al., 2015; Rabin et al., 2011), in the emotional dimension with emotional dysregulation (Wiwatowska et al., 2025) or unsuccessful down‐regulation of task‐related negative affect (e.g., Duru et al., 2023; Eckert et al., 2016) and, in the behavioural dimension, with failure to prepare the learning environment to avoid distraction (e.g., Chen et al., 2016; Senécal et al., 2003).

Procrastination is associated with lower academic achievement (e.g., Kim & Seo, 2015; Morris & Fritz, 2015), especially for students who procrastinate habitually (i.e., who have high levels of trait procrastination), because they exhibit more procrastination episodes (i.e., state procrastination; cf. Bäulke et al., 2021). This group is most in need for (targeted) interventions and empirical evidence suggests that appropriate interventions can change habitual procrastination (e.g., van Eerde & Klingsieck, 2018).

While earlier studies focused on negative emotional consequences of procrastination in the form of shame, regret or anxiety (Ferrari et al., 2009; Giguère et al., 2016; Wang, 2021), more recent studies also include emotional antecedents of procrastination. These findings demonstrate that procrastination is preceded by anxiety, shame, guilt and hopelessness (Oflazian & Borders, 2023; Rahimi et al., 2023), fear of failure (Danne et al., 2024) and general negative affect (Bruns et al., 2024). Thus, when designing measures to reduce procrastination, researchers should monitor emotional consequences of these measures to avoid unintended effects on procrastination.

As current programmes against procrastination understand it as a self‐regulation failure (see van Eerde & Klingsieck, 2018), they focus on changing aspects of cognition, emotion and behaviour. Although effective to a certain degree, heterogeneity of effects (see van Eerde & Klingsieck, 2018) indicate the need for additional measures such as changes in the learning environment (see Svartdal et al., 2020). This may represent a shift from treating individuals' procrastination to preventing it on a larger scale by changes in the context (see Biglan, 2018; Frieden, 2010), and it corresponds to contextual approaches in educational psychology, which focus on creating favourable environments (e.g., Dignath & Büttner, 2018).

Correlational findings at different levels of study programmes demonstrate that situational factors are related to procrastination, as shown by the following examples. On a macro level, curriculum design has been identified as a potential antecedent. Loosely structured curricula were associated with higher procrastination than stricter curricula (Nordby et al., 2017). On a meso level, different aspects of classroom management go along with higher procrastination, such as poor sequencing of tasks (Bäulke & Dresel, 2023) or a teaching style that thwarts basic psychological needs (Codina et al., 2024). On a micro level, academic tasks are more likely to be procrastinated when they are perceived as ambiguous (Wieland et al., 2022) or have no deadline (or lenient ones; Ariely & Wertenbroch, 2002; Milgram et al., 1992). Social factors have been studied far less, but extant results show lower procrastination when social support is high (Yang et al., 2023), when peer distraction is low (Chen et al., 2016) and when social norms suggest starting assignments promptly (Ackerman & Gross, 2005).

### Group work and effort gains

In Educational Psychology, Social Interdependence Theory and the concept of cooperative learning suggest that interdependence between group members enhances individual effort and performance (Johnson et al., 2007; Johnson & Johnson, 2002). Meta‐analyses support this suggestion (Roseth et al., 2008; Springer et al., 1999). Interdependence is given when “individuals share common goals and each individual's outcomes are affected by the actions of the others” (Johnson & Johnson, 2015; p. 857).

Similar findings from Social Psychology show that indispensability perceptions can lead to gains in individual effort and performance in group as compared to individual work (Torka et al., 2021; Weber & Hertel, 2007). The Team member Effort Expenditure Model (TEEM, Torka et al., 2021) offers an explanation for this effect. Based on expectancy × value theories of motivation, TEEM postulates that group as compared to individual work leads to effort gains, if any of its three components are more pronounced in group as compared to individual work. These three components comprise (i) the relationship between own behaviour and the outcome that results from the performance, such as monetary incentives or social recognition (i.e., the expectancy component), (ii) the value of the required behaviour or its consequences and (iii) the cost/benefit ratio of effort expenditure. According to the model, indispensability perceptions in group work are tantamount with a perceived strong relationship between individual behaviour and the outcome, thus, increasing the expectancy component. Consequently, individuals exhibit greater effort and better performance as compared to working alone. Meta‐analyses support these assumptions across settings and task types (Torka et al., 2021; Weber & Hertel, 2007; see also Hüffmeier et al., 2022). This demonstrates that perceived indispensability in group work is a predictor for effort gains in groups.^2^

Indispensability perceptions can result from different combinations of group task structure and relative member ability. In group work with a conjunctive task structure, the group member with the lowest relative ability perceives their own contribution as indispensable and thus, exhibits effort gains (Torka et al., 2021; Weber & Hertel, 2007). In group work with an additive task structure, this effect has not always been found (Hertel et al., 2000, 2003), presumably because indispensability perceptions are less pronounced in additive as compared to conjunctive group work. In additive group work, the group performance depends on all members, including the member with the lowest ability. This implies that weak individual contributions can be (at least partly) compensated by stronger group members. Indispensability perceptions may, thus, be less pronounced than during conjunctive group work.

Most group tasks are assumed to resemble additive tasks (e.g., Kelley et al., 2003), which underlines the practical relevance of this task structure. Conjunctive group tasks, on the contrary, are of higher theoretical interest, because indispensability perceptions as the relevant mediator should be especially pronounced in this type of group work. Studies on the effect of group work on procrastination should, therefore, consider both conjunctive and additive group work in comparison to individual work to enhance the usefulness of the findings.

However, the question still is whether group as compared to individual work may also help to reduce procrastination. Perceiving the own contribution as indispensable for the other group members may activate prosocial motives (cf. Priniski et al., 2019; Yeager et al., 2014) and/or altruism norms (cf. Henrich & Mathukrishna, 2021; Penner et al., 2005), that is, motives and norms relating to having a positive impact on others and not causing harm. Procrastinating the own contribution would jeopardize the group product and may represent a norm transgression, while implementing the intended action (and contributing to the group task) would support the group product and represent norm compliance. Indispensability perceptions during group work should, thus, facilitate the implementation of an intended action, especially for high trait‐procrastinators, because they have reported high levels of self‐consciousness regarding their procrastination (Ferrari, 1991a) and high concerns about their public image (Ferrari, 1991b).

First evidence exists showing how group work can reduce procrastination. The aforementioned vignette study (Koppenborg et al., 2024) showed lower procrastination in group as compared to individual work situations when relative ability was low and task structure was conjunctive or additive. In a field experiment investigating consecutive group work (Koppenborg & Klingsieck, 2022), fellow group members depended on the timely and accurate contribution of the participants to start their own part of the work (i.e., the participants worked first and believed that their fellow members would follow one after another). This interdependence led to reduced procrastination of participants as compared to individual work. However, it may be that participants in this study did not perceive a shared group goal, which is a defining characteristic of group work (see Wiese et al., 2015). Thus, it remains unclear whether the extant interdependence resulted in participants' perception that they worked on a group task.

### Group work and performance gains and changes in affect

Conjunctive and additive group work also lead to higher individual performance as compared to individual work (Torka et al., 2021; Weber & Hertel, 2007). This should be especially relevant for students who procrastinate habitually (i.e., who have high levels of trait procrastination), because these students typically exhibit decrements in academic performance (Kim & Seo, 2015; Morris & Fritz, 2015). Lower performance may occur because, as compared to non‐procrastinators, procrastinators start to work on assignments later (e.g., Michinov et al., 2011), employ study skills to a lesser degree (Svartdal et al., 2022) and submit their results later (e.g., Klingsieck & Fries, 2012).

Perceiving the own contribution to a group product as indispensable is associated with increased positive affect (Hertel et al., 2018) and task enjoyment (Hertel et al., 2003). This is consistent with theoretical assumptions from Work and Organizational Psychology, which postulate a positive relationship between task significance (i.e., high impact of the task on other peoples' lives) and job satisfaction (Hackman & Oldham, 1975). However, it is conceivable that an indispensable contribution may also be perceived as a burden, if an individual group member fears to disappoint the other group members by not showing a good individual performance (cf. Weber & Hertel, 2007). Thus, while increasing positive affect, indispensability perceptions may simultaneously increase negative affect. This notion is consistent with the theoretical assumption that positive and negative affect are independent of each other (see Watson & Tellegen, 1985), and initial evidence from a vignette study also points into this direction (Koppenborg et al., 2024).

Following the mood‐repair hypothesis (Sirois & Pychyl, 2013), students procrastinate assignments to avoid task‐related negative affect, such as anxiety (Pychyl et al., 2000). Interventions aiming to reduce procrastination may prove counterproductive if they lead to higher negative or lower positive task‐related affect. Thus, beyond task performance, this study also investigated effects of group work on positive and negative affect.

### Present study and hypotheses

This study investigated whether conjunctive and additive group work as compared to individual work (i) reduces procrastination of an academic assignment, (ii) increases performance and (iii) how these types of group work influence positive and negative task‐related affect. Indispensability perceptions were explored as a potential underlying variable.^3^ Because students who procrastinate habitually are especially in need of effective interventions, the study focuses on the group of high trait‐procrastinators. Based on previous work on conjunctive group work, effort, performance and positive affect (Johnson & Johnson, 2002; Torka et al., 2021; Weber & Hertel, 2007) and initial evidence regarding the relationship to procrastination (Koppenborg et al., 2024; Koppenborg & Klingsieck, 2022), we formulate the following hypotheses^4^: When their relative ability is low, participants in conjunctive group as compared to individual work exhibit lower task procrastination (H1a), higher individual task performance (H1b) and higher task‐related positive affect (H1c).

In additive group work, the contribution of the weakest member can be compensated (at least partially) by stronger members, rendering perceptions of indispensability and potential effort gains lower as compared to conjunctive group work (see Hertel et al., 2000, 2003). We explore this by formulating research questions (RQ): When their relative ability is low, do participants in additive group work as compared to individual work differ regarding their task procrastination (RQ1a), task performance (RQ1b) and task‐related positive affect (RQ1c)?

Because task‐related negative affect can be an important antecedent of procrastination episodes (see Eckert et al., 2016; Rahimi et al., 2023), we also explore the effects of both types of group work on negative affect by formulating the following research questions: When group member relative ability is low, is there a difference in negative task‐related affect between conjunctive group work and individual work (RQ2a) and between additive group work and individual work (RQ2b)? Finally, we explore the role of indispensability perceptions as a variable that potentially underlies the hypothesized effects. Accordingly, we assume that, when group member relative ability is low, their indispensability perceptions are higher in conjunctive as compared to additive group work (H2).

## METHOD

Students with a high level of trait procrastination completed a typical academic task (i.e., compiling a bibliography) in one of three experimental conditions. In the individual condition, participants worked alone. In both group work conditions, participants were led to believe that they were part of a group with two other students. Participants in the conjunctive group work condition were informed that their group performance was determined by the poorest individual contribution. Participants in the additive group work condition were informed that their group performance was determined by the average of all three contributions. In all three conditions, participants were led to believe that they had low ability in the assignment. Self‐ratings on procrastination, task‐related affect and indispensability, as well as objective performance measures were compared between conditions.^5^

### Participants

An a priori power analysis indicated an overall minimum sample of *N* = 150 participants (*n* = 50 participants in each condition; assuming *α* = .05, and 1 − *β* = .9; the smallest assumed effect was *d* = .6; for details, see ESM). The experiment was approved by the local ethics commission (decision No. GEKTUDO_2024_10). According to the preregistration, data collection was planned to take place over the course of 2–4 weeks during a regular semester until the intended sample size would be reached. Three aspects of our realized data collection are noteworthy. First, data collection took in fact longer, because it was difficult to acquire participants for the screening, and to motivate participants to provide their contact information, once they were included in the study. Thus, data collection took place for 6 weeks during the winter semester 2024 and it continued for 4 weeks during the summer semester 2025. Second, because participants could decide to end their participation during the instruction and not provide their contact information, we expected that unequal cell samples could occur. To avoid this, and in accordance with the preregistration, random assignment to conditions was adapted each week (i.e., it was weighed depending on the current samples in each condition). However, recruitment numbers varied each week in an unpredictable way. This resulted in one condition (i.e., individual work) with a larger proportion of participants who took part in summer of 2025, and the two group work conditions with a larger proportion of participants who took part in winter of 2024. This was considered in data analysis and interpretation of the results.

After providing informed consent, a total of 1535 participants from different German universities completed an online screening survey (1129 female, 15 diverse, nine did not report their gender). In accordance with the preregistered procedure, students were invited to take part in the main study only if they met the inclusion criteria (see below). A total of *N* = 218 (150 female, three diverse) participated in the main study by providing their contact information (i.e., email address and phone number). Table 1 provides a description of the sample (for a description of excluded participants, see Table E1 in the ESM). Study programmes comprised diverse fields such as humanities, natural sciences, social sciences and economics. Sample sizes were smaller for some dependent variables (see Table 3), because not all participants provided ratings for these variables.

**TABLE 1:** Description of the sample.

	Number of participants in experiment			
	Total	Male	Female	Diverse
Total	218	65	150	3
Experimental condition				
Individual	81	25	56	1
Conjunctive	71	21	50	2
Additive	66	22	44	0
Age				
18–21 years	99			
22–24 years	49			
25–27 years	45			
28 years and above	25			
Programmes				
Bachelor's	167			
Master's	44			
No answer	7			
Year of study				
First	82			
Second	55			
Third	39			
Above third	35			
No answer	7			

### Task and reward

The task was to compile a 10‐entry bibliography in accordance with the *Publication Manual of the American Psychological Association* (2020; hereafter referred to as APA guidelines) and its submission to the experimenter within 10 days. During this time, participants could work on the bibliography according to their own schedules. Participants were randomly assigned to the three experimental conditions (individual work vs. conjunctive group work vs. additive group work), and task instructions varied depending on the condition. To realize high comparability of our procedure with academic grading schemes (i.e., less money/worse grades for lower performance quality) and, thus, a high ecological validity, our reward scheme was analogous to such procedures. Participants' compensation depended on the quality of their submitted bibliography (i.e., the number of errors). For flawless bibliographies, participants could receive €15. This reward decreased depending on the number of errors in the assignment. To offer a stronger incentive for participation, each participant would receive an additional compensation of €5 upon submitting the bibliography regardless of its quality.

### Variables and measures

#### Inclusion criteria

Participants were included in the study if they reported high levels of trait procrastination, had no prior experience with working with APA guidelines and had not participated in similar studies before.

##### Trait procrastination

The General Procrastination Scale was used in its shortened German version (GPS‐K; Klingsieck & Fries, 2012; *α* = .86; nine items; for example, “I often find myself performing tasks that I had intended to do days before”) to assess trait procrastination on a 4‐point scale ranging from 1 (*very untypical*) to 4 (*very typical*). Students with a score of 2.78 and higher were invited to participate in the study. Based on previous studies (Koppenborg & Klingsieck, 2022), this cut‐off should include one third of the screened participants with the highest trait procrastination.

##### Experience with APA guidelines

Participants' experience with APA guidelines was assessed with one item (“Have you ever compiled a bibliography according to the guidelines of the American Psychological Association [APA]?”) that was rated on a 3‐point scale (“yes”, “no”, “I don't know”). Only students with no prior experience (i.e., answering with “no”) were invited to participate in the study.

##### Participation in similar studies

Prior participation was assessed with one item (“Have you participated in a similar study during the last 24 months?”) that was rated on a 3‐point scale (“yes”, “no”, “I don't know”). Students could only participate in the study if they had not taken part in a similar study before (i.e., answering with “no”).

#### Independent variable

##### Task structure

To realize the experimental manipulation, instructions on the task were systematically varied (see Table 2). In all three conditions, the instructions included details about the bibliography, the deadline and the reward. To induce perceptions of low ability among the participants, instructions stated that the task was typically difficult for the participants. In the individual work condition, participants received no further information. In the two group work conditions, participants were instructed depending on either the conjunctive or additive task structure (see Table 2). The experimental conditions were realized by instruction only, no real groups were formed. The experimenter was blind to the conditions of individual participants. To increase the credibility of the instructions in the two group conditions, the participants were asked to provide a short, written greeting for the other group members. They also received fictitious and generic greetings from their group members (e.g., “Hello, nice to work with you!”), which were identical for all participants. The participants in both group conditions did not receive additional information about the other group members.

**TABLE 2:** Instructions on the assignment for each experimental condition.

	Individual work condition	Conjunctive group work condition	Additive group work condition
Relevance	By completing the assignment, you can earn (up to) €20.	By completing the assignment, you can earn (up to) €20.	
Task structure	You will work on the assignment *on your own*. For your own compensation, the quality of your assignment will be considered	You will work on the assignment on your own, but you are part of a group with *two other students*. For your own compensation, the quality of all three assignments will be considered.	
Task aversiveness	You will compile a bibliography according to the APA rules.	Each group member will compile a bibliography according to the APA rules.	
Task aversiveness	The bibliography has to include 10 entries.	Each bibliography has to include 10 entries	
Relevance	You will receive €5 for submitting the assignment, regardless of its quality. Your additional compensation will depend on the number of errors in the assignment.	Each group member will receive €5 for submitting their assignment, regardless of its quality. Your additional compensation will depend on the number of errors in the assignment.	
Task structure	For every accurate entry, one point will be given. Example: With seven accurate entries in your bibliography, you will receive seven points. Every point corresponds to €1.50 that you will receive. No points will be given for a bibliography that is submitted after the deadline.	The additional compensation for all group members will be determined by *the bibliographies with the most errors (i.e., the worst bibliography).* For every accurate entry in the worst bibliography, one point will be given. Example: With seven accurate *entries in the worst bibliography*, each of you will receive seven points. Every point corresponds to €1.50 that each of you will receive. No points will be given for a bibliography that is submitted after the deadline.	The additional compensation for all group members will be determined by the *average number of errors across the three bibliographies.* For every accurate entry, one point will be given. Example: With an *average* of seven accurate entries across the three bibliographies, each of you will receive seven points. Every point corresponds to €1.50 that each of you will receive No points will be given for a bibliography that is submitted after the deadline.
Task structure		This means that the bibliography with the *lowest number of points* determines the additional compensation for every group member.	This means that the *average number of points* across the three bibliographies determines the additional compensation for every group member.
Relevance	Therefore, the total compensation for you will range from €5 to €20.	Therefore, the total compensation for each of you will range from €5 to €20.	
		You will be automatically assigned to your group. On the next pages, you will find information on the other group members.	
Relative ability	Our previous studies have shown that students of your subject and your college year find it more difficult to succeed in this assignment, compared to students of other subjects. However, if you put effort into it, you are likely to succeed in working on the assignment.		
Relative ability		The other group members are: Psychology students. Students of this subject are often quite good at this assignment.	

#### Dependent variables

##### Task procrastination

The first dimension of the Academic Procrastination State Inventory (Patzelt & Opitz, 2014) was used in its German shortened version (cf. Koppenborg et al., 2024; *α* = .78). The scale comprises only four items to assess state procrastination on a 5‐point scale ranging from 1 (*never*) to 5 (*constantly*). Items were adapted to the assignment (e.g., “I did so many other things that there was insufficient time left to work on the bibliography”).

##### Task performance

The compensation paid to participants was used as a measure of performance. Rules for compensation were identical in all three conditions, following the rules in the individual work condition (i.e., in the two group work conditions, the actual compensation differed from what had been described to participants in the instructions, and participants were debriefed about their actual compensation after the experiment had finished). Thus, participants in all three conditions received €5 for submitting their bibliography, regardless of its quality (i.e., regardless of the number of errors). Additionally, for every correct entry in their individual bibliography, each participant received an additional €1.5. Thus, each participant could reach a maximum of €20. For an entry to count as correct, it had to exactly accord with APA guidelines (for details, see ESM). Thus, the actual payment also served as performance measure.

##### Positive and negative affect

Participants' positive and negative affect was assessed with the 20 items of the Positive and Negative Affect Schedule (PANAS; Watson et al., 1988) in its German version (Krohne et al., 1996; .84 < *α* < .85). The PANAS items (e.g., “nervous”, “excited”) were rated on a 5‐point scale ranging from 1 (*not at all*) to 5 (*extremely*). Participants' affect was assessed at three points in time. First, before the instructions, affect regarding academic tasks in general was assessed to ensure comparability of participants between conditions regarding these variables. Second, affect with regard to compiling the bibliography was assessed directly after the instruction (prospective affect), and third, after its submission (retrospective affect).

##### Perceived indispensability

To explore indispensability perceptions as a potential underlying variable in the two group work conditions, three items were used, (e.g., “How dispensable or indispensable is your contribution for the group result?”; *α* = .66; for item formulations, see ESM) that were rated on a 5‐point scale ranging from 1 (*completely dispensable*) to 5 (*completely indispensable*).

#### Explorative variables, perceived ability and control variables

##### Duration until submission and time on task

Duration until submission (i.e., time between receiving the materials and submitting the bibliography) and time on task (i.e., time between starting and submitting the bibliography) were assessed. To allow for the assessment of the latter variable, the participants reported the exact dates and times when they had begun compiling the bibliography.

##### Perceived ability

To verify that participants had processed the information on their relative ability, perceived ability was assessed with one item (“How do you judge your skill to succeed in the task? [compared to the other group members]?”) Participants rated this item on a 5‐point scale ranging from very low (1) to very high (5).

##### Control variables

Participants' self‐ratings were assessed for (1) preference for team work, (2) social comparison and (3) extrinsic motivation (for details, see ESM).

### Procedure

**FIGURE 1:**
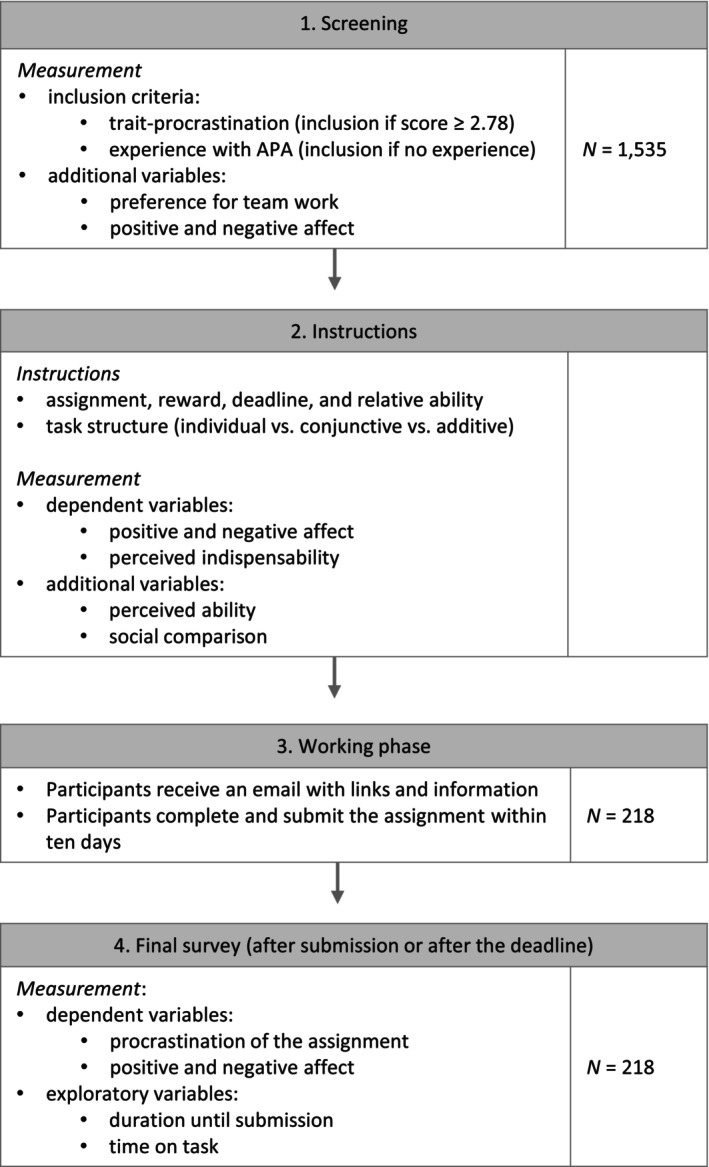
Four‐step screening and field experiment.

Figure 1 presents the four‐step procedure for our field experiment. (1) The potential participants completed an online screening survey to assess the inclusion criteria and their preference for group work. (2) The included participants were randomly assigned to one of the three experimental conditions. They read the respective instructions and provided ratings on perceived ability. The participants in the group conditions also provided ratings on perceived social indispensability and on social comparison. Further, they provided a short greeting to their (fictitious) group members. Finally, participants in all conditions were asked to provide ratings on positive and negative affect regarding the experimental task and to provide their contact information. (3) The following Monday, the working phase started with an email that was sent to participants, containing (a) a link to a video tutorial on compiling the bibliography in APA style, (b) links to the 10 research articles to be included in the bibliography and (c) a document in which to compile the bibliography. During the next 10 days, the participants could complete and submit their bibliography according to their own schedules or preferences. (4) Within 2 h of submission, they received a link to a final survey by text message to assess task procrastination. The participants who did not submit their bibliography after 5 days received a reminder email. The participants who did not submit their assignment after 10 days nevertheless received an invitation to the survey after the deadline. Submissions were evaluated, and participants received a debriefing email and their compensation.

### Manipulation checks

All participants had a trait procrastination score of 2.78 or higher, had no prior experience with APA guidelines and had not participated in similar studies before. Five separate between‐subjects ANOVAs showed no significant differences across experimental conditions regarding the five control variables (all *ps* > .05). Three separate independent *t*‐tests showed that, in each of the three conditions, mean ratings for perceived ability were significantly lower than the mean point of the scale (4, all *ps* < .05). This confirmed that participants had adequately processed the instructions and had perceived their own ability as low or medium. Outliers were found only for negative task‐related affect and were removed from analysis (see below).

Regarding the sampling period, between‐subjects ANOVAs were calculated within each experimental condition to analyse whether sampling period affected our dependent variables (see Table E2 in the ESM for descriptive statistics). Positive and negative affect did not differ between both periods. Task procrastination was significantly lower in the summer as compared to the winter period in individual work and additive group work. Task performance was significantly higher in the summer as compared to the winter period (see Table E3 in the ESM for all test statistics). Due to the unpredictable participation rate, sample sizes in each cell varied considerably (see Table 3).

**TABLE 3:** Number of participants across sampling periods (for the dependent variable of task procrastination).

	Number of participants		
	Individual work	Conjunctive group work	Additive group work
Winter period	27	55	45
Summer period	37	3	10

Thus, the summer period was overrepresented in the individual work condition, while the winter period was overrepresented in the two group work conditions. According to our hypothesis, procrastination should be higher in individual work as compared to the two group work conditions. Thus, the unequal sample sizes across conditions and sampling periods influenced the data in a direction opposite to our hypothesis. This was taken into account in additional, unplanned analyses of task procrastination (see below).

### Study design and data analyses

The following analyses were preregistered beforehand. For all variables, outliers were analysed using the interquartile range with a multiplier of 3, and sensitivity analyses were conducted if outliers were found. For Hypotheses 1a to 1c and all Research Questions, ordinary least squares regressions were calculated with experimental condition as a predictor, while controlling for preference for group work. To test Hypothesis 2 regarding perceived indispensability as a potential underlying variable, in a first step, between‐subjects ANOVA was calculated with a planned contrast using a bootstrapped *t*‐test. To control for social comparison, in a second step, ordinary least squares regression was calculated with perceived indispensability as criterion and social comparison as predictor. The residuals of this calculation represent scores of perceived indispensability without the variance that is due to social comparison. A second between‐subjects ANOVA was then calculated with these residuals as the dependent variable. According to the preregistration, a potentially significant result of this analysis would be analysed with a planned contrast using a bootstrapped *t*‐test. Such a significant result would indicate that differences in perceived indispensability are consistent with mediation. For all analyses, the significance level was set at .05.

Due to the unplanned second sampling period (see above), additional analyses were conducted that had not been preregistered beforehand. These served to analyse whether the sampling period affected our dependent variables and to account for this potential influence by including sampling period as a control variable.

## RESULTS

Descriptive statistics of manipulation checks and all control variables, dependent variables and exploratory variables are shown in Table 4.

**TABLE 4:** Descriptive statistics of all manipulation checks, control variables, dependent variables and exploratory variables (outliers excluded).

	Individual work			Conjunctive group work			Additive group work		
	*M*	*SD*	*N*	*M*	*SD*	*N*	*M*	*SD*	*N*
Manipulation check									
Perceived ability	3.70	.94	81	3.41	.92	71	3.29	.99	66
Control variables									
Trait procrastination	3.21	.42	81	3.20	.03	71	3.22	.35	66
Positive affect academic tasks	2.73	.69	81	2.62	.74	70	2.69	.79	66
Negative affect academic tasks	2.29	.85	81	2.40	.94	70	2.41	.89	66
Preference for teamwork	2.52	1.10	81	2.57	1.24	71	2.57	1.12	66
Extrinsic motivation	4.19	.81	81	3.95	1.10	71	4.16	.95	66
Social comparison				3.70	1.22	71	3.77	1.05	66
Dependent variables									
Perceived indispensability				3.98	.70	71	3.85	.91	66
Task procrastination	3.04	1.10	64	2.69	.93	58	3.00	1.04	53
Task performance	5.08	5.76	81	6.13	6.47	71	5.49	5.83	66
Positive affect (prosp.)	2.99	.79	81	2.83	.76	71	2.91	.60	66
Positive affect (retrosp.)	2.58	.90	64	2.69	1.03	58	2.69	.77	53
Negative affect (prosp.)	1.42	.48	80	1.57	.51	68	1.69	.66	64
Negative affect (retrosp.)	1.61	.66	63	1.64	.75	57	1.65	.70	52
Exploratory variables									
Duration until submission [hh:mm]	136:47	80:26	43	145:50	75:52	43	158:03	77:20	38
Time on task [hh:mm]	27:35	56:04	41	25:50	43:57	38	19:25	32:24	38

*Note*: Sample sizes vary between dependent variables, because not all participants filled out the final survey on task procrastination and retrospective affect (in accordance with the preregistration).

### Conjunctive group work vs. individual work

#### Task procrastination

Results revealed a non‐significant overall model fit and a significant coefficient for conjunctive group work (see Model 1 in Table 5). Two additional exploratory analyses were run. A one‐way ANOVA (conjunctive group work vs. individual work) revealed a significant effect of experimental condition on task procrastination, *F*(1, 120) = 3.71, *p* = .029, *d* = .35 (i.e., a small effect; Cohen, 1988). Next, a hierarchical ordinary least squares regression was calculated to account for sampling period. In step 1, task procrastination was regressed on preference for group work and sampling period. The overall fit of this model was non‐significant and both coefficients were non‐significant (see Model 2a). In step 2, experimental condition (i.e., conjunctive group work vs. individual work) was added as a predictor. The overall fit of this model was significant and the coefficient for conjunctive group work was negative and significant (see Model 2b). Taken together, results are inconclusive regarding H1a, with some evidence suggesting that conjunctive group work results in lower task procrastination as compared to individual work and some evidence suggesting no difference.

**TABLE 5:** Results of regression analyses with task procrastination as criterion.

	Model 1		Model 2a		Model 2b	
	*β*	*p*	*β*	*p*	*β*	*p*
Preference for group work	.11	.21	.10	.27	.11	.20
Sampling period^a^			−.03	.75	−.19*	.08
Conjunctive group work^b^	−.18*	.048			−.29**	.009
*R* ^*2*^	.04		.01		.07	
*F*	2.66		.69		7.07	
Overall model fit *p*	.074		.51		.01	

^a^ Dichotomous variable for sampling period (1 = summer period, 0 = winter period).

^b^ Experimental condition (1 = conjunctive group work, 0 = individual work).

* *p* < .05.

** *p* < .01.

#### Task performance

Results revealed a non‐significant overall model fit and a non‐significant coefficient for conjunctive group work (see Model 3 in Table 6). Thus, H1b was not supported.

**TABLE 6:** Results of regression analyses for conjunctive group work vs. individual work with performance, positive affect and negative affect as criterion.

	Model 3		Model 4a		Model 4b		Model 5a		Model 5b	
	*β*	*p*	*β*	*p*	*β*	*p*	*β*	*p*	*β*	*p*
Preference for group work	−.08	.33	−.17*	.041	−.04	.68	−.06	.45	.11	.24
Conjunctive group work^a^	.09	.28	−.11	.19	.06	.51	.16*	.03	.03	.77
*R* ^*2*^	.01		.04		.01		.03		.01	
*F*	1.04		2.91		.29		2.09		.79	
Overall model fit *p*	.36		.06		.75		.13		.46	

*Note*: Criteria used in regression analyses: Task performance (Model 3), positive task‐related affect (prospective and retrospective, Models 4a and 4b), negative task‐related affect (prospective and retrospective, Models 5a and 5b).

^a^ Experimental condition (1 = conjunctive group work, 0 = individual work).

* *p* < .05.

#### Positive and negative task‐related affect (prospective and retrospective)

Positive and negative affect regarding the experimental task were assessed after receiving task instructions (i.e., prospectively) and after finishing the task (i.e., retrospectively).^6^ For positive affect, results of both analyses revealed non‐significant overall model fits and non‐significant coefficients for conjunctive group work (see Model 4a for prospective, and Model 4b for retrospective positive affect). Thus, H1c was not supported.

For prospective negative affect, results revealed a significant overall model fit and a coefficient for conjunctive group work that was significant and positive (see Model 5a). This indicates higher negative task‐related affect in conjunctive group work as compared to individual work. For retrospective negative affect, results revealed a non‐significant overall model fit and a non‐significant coefficient for additive group work (see Model 5b). Taken together, results are inconclusive regarding RQ2a, with some evidence suggesting that negative task‐related affect differs between conjunctive group work and individual work.

### Additive group work vs. individual work

#### Task procrastination

Results revealed a non‐significant overall model fit and a non‐significant coefficient for additive group work (see Model 6 in Table 7). In view of RQ1a, task procrastination does not differ between additive group work and individual work.

**TABLE 7:** Results of regression analyses for additive group work vs. individual work with procrastination, performance, positive affect and negative affect as criterion.

	Model 6		Model 7		Model 8a		Model 8b		Model 9a		Model 9b	
	*β*	*p*	*β*	*p*	*β*	*p*	*β*	*p*	*β*	*p*	*β*	*p*
Preference for group work	.13	.16	−.09	.29	.10	.22	−.03	.72	−.04	.65	.08	.43
Additive group work^a^	−.03	.79	.04	.65	−.05	.52	.07	.45	.23**	.003	.07	.50
*R* ^*2*^	.02		.01		.01		.01		.06		.01	
*F*	1.02		.67		.94		.35		4.14		.57	
Overall model fit *p*	.36		.51		.39		.71		.02		.57	

*Note*: Criteria used in regression analyses: Task procrastination (Model 6), task performance (Model 7), positive task‐related affect (prospective and retrospective, Models 8a and 8b), negative task‐related affect (prospective and retrospective, Models 9a and 9b).

^a^ Experimental condition (1 = additive group work, 0 = individual work).

** *p* < .01.

#### Task performance

Results revealed a non‐significant overall model fit and a non‐significant coefficient for additive group work (see Model 7). In view of RQ1b, task performance does not differ between additive group work and individual work.

#### Positive and negative task‐related affect (prospective and retrospective)

Regarding positive affect, results revealed non‐significant overall model fits and non‐significant coefficients for additive group work (see Model 8a for prospective, and Model 8b for retrospective positive affect). In view of RQ1c, it appears that positive task‐related affect does not differ between additive group work and individual work. For prospective negative affect, results revealed a significant overall model fit and a coefficient for additive group work that was significant and positive (see Model 9a).^7^ This indicates higher negative task‐related affect in additive group work as compared to individual work. For retrospective negative affect, results revealed a non‐significant overall model fit and a non‐significant coefficient for additive group work (see Model 9b). Taken together, results are inconclusive regarding RQ2b, with some evidence suggesting that negative task‐related affect differs between additive group work and individual work and other evidence suggesting a non‐difference.

#### Duration until submission and time on task in conjunctive and additive group work

Analyses revealed non‐significant overall model fits and non‐significant coefficients for conjunctive and additive group work for both exploratory variables.

### Perceived indispensability

A between‐subjects ANOVA revealed no significant difference in perceived indispensability between conjunctive and additive group work. A second between‐subjects ANOVA with the residuals of perceived indispensability, that is, the scores of perceived indispensability without the variance explained by social comparison, revealed no significant difference between conjunctive and additive group work. Thus, H2 was not supported.

## DISCUSSION

### Summary

This registered field experiment investigated the effects of conjunctive and additive group work as compared to individual work on task procrastination, task performance and task‐related positive and negative affect in a sample of students with high levels of trait procrastination. The preregistered analyses showed no effect of conjunctive group as compared to individual work on task procrastination, whereas two additional exploratory analyses showed lower task procrastination in conjunctive group work. The latter result converges with theoretical accounts and empirical findings showing gains in individual effort when the own contribution is indispensable to the other group members (Johnson et al., 2007; Torka et al., 2021); and it converges with and extends previous findings showing lower procrastination in conjunctive group work (Koppenborg et al., 2024) and interdependent group work (Koppenborg & Klingsieck, 2022). No effect of conjunctive group work on task performance was found. Descriptive statistics, however, indicate the direction of the expected effect. Further, no effect on positive affect was found, nor on negative affect when assessed retrospectively. However, negative affect was higher in conjunctive group work when assessed prospectively. These results diverge from findings of previous studies, which found higher task performance in consecutive group as compared to individual work (Koppenborg & Klingsieck, 2022), and higher positive and higher negative task‐related affect in conjunctive group work as compared to individual work (Koppenborg et al., 2024).

Overall, our results concerning procrastination and performance might be explained with the mood‐repair hypothesis, which states that procrastination serves to avoid negative task‐related emotions (e.g., Sirois & Pychyl, 2013). Consistent with this, procrastination is often preceded by anxiety, shame and guilt (e.g., Rahimi et al., 2023). Along these lines, perceptions of indispensability in conjunctive and additive group work may have resulted in fears to disappoint other group members with a poor individual performance, thus explaining higher levels of negative affect in in both group work conditions (when assessed prospectively). Possibly, the beneficial effects of conjunctive and additive group work on procrastination and performance have been counteracted, at least to some degree, by higher levels of negative affect.

To complicate matters further, data collection took place during two time periods, not within one (as initially preregistered). Both periods differed regarding task procrastination, the main dependent variable, that is, task procrastination was lower in the summer period as compared to the winter period. More importantly, sample sizes for the winter and the summer period differed substantially across conditions, because allocation of participants to experimental conditions was adapted in each week, while recruitment success was hard to predict. This confound affected the data in an opposite direction to our hypothesis, which could be another reason why our preregistered analyses failed to find the expected effects. When including sampling period as control variable, however, results showed lower task procrastination in conjunctive group work as compared to individual work (in support of our assumption).

### Limitations and future research

In addition to the aforementioned limitation concerning our sampling strategy, limitations pertain to our measures and to our final sample. Although our a priori power analysis was based on empirical findings of similar studies, our overall sample size may have been insufficient for some dependent variables because possible effects may have been smaller as expected. This is suggested by descriptive differences in task performance and indispensability perceptions that were aligned to our hypotheses. Further, because we were interested in the isolated effects of conjunctive and additive task structures, the two group work conditions did not include any social interactions among group members. Such interactions typically occur in real settings of group work, which may limit the ecological validity of our findings. Regarding our measures, it remains unclear whether our incentive (i.e., monetary reward) resembles grades or academic credit points and whether our results generalize to academic tasks other than compiling bibliographies. Further, internal consistency of our measure for indispensability perceptions was rather low. The current literature lacks a validated instrument to assess indispensability perceptions, which could be the subject of future research. Finally, the study focused on students with high level of habitual procrastination, while female participants were overrepresented in the sample. Results may differ with samples that include more male participants and those with lower levels of habitual procrastination.

### Theoretical and practical implications

Human behaviour is a result of personal and situational factors (Furr & Funder, 2021; Lewin, 1951). Our findings, albeit inconclusive in some regards, add to the yet scarce research on the role of situational and social factors when describing and explaining procrastination (cf. Bäulke & Dresel, 2023; Svartdal et al., 2020). Interestingly, the importance of these factors became apparent in our study when it comes to the role of negative affect. Considering that procrastination can serve to avoid negative affect (see the mood‐repair hypothesis, Sirois & Pychyl, 2013), situational interventions with the aim to reduce procrastination may prove ineffective if they entail negative affective reactions. Further, the findings add to the literature on conjunctive and additive group work. There is much evidence on gains in effort and performance due to the effects of group work and indispensability perceptions (Torka et al., 2021; Weber & Hertel, 2007). Although our results have to be interpreted with caution, they suggest that these effects may also apply to procrastination.

If corroborated by future research, our results can inspire teachers and counsellors to consider situational factors in general, and group work with perceived indispensability in particular. Because legal and ethical reasons may prohibit conjunctive group work in practice, indispensability perceptions can be induced by other means, for example, by creating groups in which each member has unique skills, roles or contributions (cf. Weber & Hertel, 2007).

Lastly, and on a more general level, the notion that situational factors are relevant for procrastination provides a chance to consider an extension of current person‐centred interventions (cf. van Eerde & Klingsieck, 2018). Complementing these with targeted situational changes may increase overall effectiveness. As shown in this contribution, group work as a situational and social factor can be a promising component of such an approach.

## AUTHOR CONTRIBUTIONS

**Markus Koppenborg:** Conceptualization; data curation; formal analysis; investigation; methodology; writing – original draft; writing – review and editing. **Joachim Hüffmeier:** Conceptualization; data curation; methodology; writing – original draft; writing – review and editing; resources. **Katrin B. Klingsieck:** Conceptualization; data curation; formal analysis; investigation; writing – original draft; writing – review and editing.

## CONFLICT OF INTEREST STATEMENT

The authors declare no conflicts of interest.

## Supporting information


Data S1:

## Data Availability

This manuscript is submitted as a registered report. Data can be made available upon request.

## Footnotes

^1^
The original Stage 1 Manuscript of this registered report can be accessed online: https://osf.io/kn7t8/?view_only=2ed6c47db80048dd85799dfc26e22076.

^2^
Apart from perceived indispensability, social comparison (see Seta, 1982) can also cause effort gains in group as compared to individual work (see Weber & Hertel, 2007). Therefore, we also assess social comparison (see the method section for more information on this control variable).

^3^
The nature of the field experiment prohibits a large sample, which would be necessary to conduct adequately powered mediation analyses. Therefore, indispensability perceptions are not investigated as a mediator variable. Instead, we explored whether indispensability perceptions are more pronounced in conjunctive as compared to additive group work, which would be consistent with mediation.

^4^
To facilitate understanding of our hypotheses and research questions, we use the word “task” instead of the preregistered word “assignment” (e.g., “task procrastination”, instead of “procrastination of the assignment”). This change of words does not change the meaning of hypotheses or research questions.

^5^
Indispensability perceptions were only compared between the conjunctive and additive group work conditions because indispensability perceptions are not relevant for individual work.

^6^
Regarding negative affect, four cases (prospective) and two cases (retrospective) were identified as outliers, and, thus, excluded from analysis. Sensitivity analyses were conducted that included all outliers. Results did not differ from the results reported above.

^7^
Three cases for prospective affect, and two cases for retrospective affect were identified as outliers, and, thus, excluded from analysis. Sensitivity analyses were conducted that included all outliers. Results did not differ from the results reported above.

## References

[bjep70069-bib-0001] Ackerman, D. S. , & Gross, B. L. (2005). My instructor made me do it: Task characteristics of procrastination. Journal of Marketing Education, 27(1), 5–13. 10.1177/0273475304273842

[bjep70069-bib-0002] Ariely, D. , & Wertenbroch, K. (2002). Procrastination, deadlines, and performance: Self‐control by precommitment. Psychological Science, 13(3), 219–224. 10.1111/1467-9280.00441 12009041

[bjep70069-bib-0003] Bäulke, L. , Daumiller, M. , & Dresel, M. (2021). The role of state and trait motivational regulation for procrastinatory behavior in academic contexts: Insights from two diary studies. Contemporary Educational Psychology, 65, 101951. 10.1016/j.cedpsych.2021.101951

[bjep70069-bib-0004] Bäulke, L. , & Dresel, M. (2023). Higher‐education course characteristics relate to academic procrastination: A multivariate two‐level analysis. Educational Psychology, 43(4), 263–283. 10.1080/01443410.2023.2219873

[bjep70069-bib-0005] Biglan, A. (2018). The ultimate goal of prevention and the larger context for translation. Prevention Science, 19(3), 328–336. 10.1007/s11121-016-0635-6 26910318 PMC4996764

[bjep70069-bib-0006] Bruns, K. , Eggert, P. , & Wolgast, A. (2024). Students' anticipated negative consequences of procrastination behavior and momentary affective experiences. Zeitschrift für Entwicklungspsychologie Und Pädagogische Psychologie, 56(4), 162–174. 10.1026/0049-8637/a000299

[bjep70069-bib-0007] Chen, B.‐B. , Shi, Z. , & Wang, Y. (2016). Do peers matter? Resistance to peer influence as a mediator between self‐esteem and procrastination among undergraduates. Frontiers in Psychology, 7, 1529. 10.3389/fpsyg.2016.01529 27752248 PMC5045931

[bjep70069-bib-0008] Codina, N. , Castillo, I. , & Pestana, J. V. (2024). Time perspectives and procrastination in university students: Exploring the moderating role of basic psychological need satisfaction. BMC Psychology, 12, 5. 10.1186/s40359-023-01494-8 38167512 PMC10759600

[bjep70069-bib-0009] Cohen, J. (1988). Statistical power analysis for the behavioral sciences. Lawrence Earlbaum Associates.

[bjep70069-bib-0010] Danne, V. , Gers, B. , & Altgassen, M. (2024). Is the association of procrastination and age mediated by fear of failure? Journal of Rational‐Emotive & Cognitive‐Behavior Therapy, 42, 433–446. 10.1007/s10942-023-00527-w

[bjep70069-bib-0011] Dignath, B. , & Büttner, G. (2018). Teachers' direct and indirect promotion of self‐regulated learning in primary and secondary school mathematics classes – Insights from video‐based classroom observations and teacher interviews. Metacognition and Learning, 13, 127–157. 10.1007/s11409-018-9181-x

[bjep70069-bib-0012] Duru, E. , Balkis, M. , & Duru, S. (2023). Procrastination among adults: The role of self‐doubt, fear of the negative evaluation, and irrational/rational beliefs. Journal of Evidence‐Based Psychotherapies, 23(2), 79–97. 10.24193/jebp.2023.2.11

[bjep70069-bib-0013] Eckert, M. , Ebert, D. D. , Lehr, D. , & Berking, M. (2016). Overcome procrastination: Enhancing emotion regulation skills reduces procrastination. Learning and Individual Differences, 52, 10–18. 10.1016/j.lindif.2016.10.001

[bjep70069-bib-0014] Ferrari, J. R. (1991a). A preference for a favorable public impression by procrastinators: Selecting among cognitive and social tasks. Personality and Individual Differences, 12(11), 1233–1237. 10.1016/0191-8869(91)90090-X

[bjep70069-bib-0015] Ferrari, J. R. (1991b). Compulsive procrastination: Some self‐reported characteristics. Psychological Reports, 68, 455–458. 10.2466/pr0.1991.68.2.455 1862177

[bjep70069-bib-0016] Ferrari, J. R. , Barnes, K. L. , & Steel, P. (2009). Life regrets by avoidant and arousal procrastinators. Why put off today what you will regret tomorrow? Journal of Individual Differences, 30(3), 163–168. 10.1027/1614-0001.30.3.163

[bjep70069-bib-0017] Frieden, T. R. (2010). A framework for public health action: The health impact pyramid. American Journal of Public Health, 100(4), 590–595. 10.2105/AJPH.2009.185652 20167880 PMC2836340

[bjep70069-bib-0018] Furr, R. M. , & Funder, D. (2021). Persons, situations, and person‐situation interactions. In O. P. John & R. W. Robins (Eds.), Handbook of personality: Theory and research. Guilford.

[bjep70069-bib-0019] Giguère, B. , Sirois, F. M. , & Vaswani, M. (2016). Delaying things and feeling bad about it? A norm‐based approach to procrastination. In F. M. Sirois & T. A. Pychyl (Eds.), Procrastination, health, and well‐being (pp. 189–212). Elsevier.

[bjep70069-bib-0020] Gustavson, D. E. , Miyake, A. , Hewitt, J. K. , & Friedman, N. P. (2015). Understanding the cognitive and genetic underpinnings of procrastination: Evidence for shared genetic influences with goal management and executive function abilities. Journal of Experimental Psychology: General, 144(6), 1063–1079. 10.1037/xge0000110 26389573 PMC4658242

[bjep70069-bib-0021] Hackman, J. R. , & Oldham, G. R. (1975). Development of the job diagnostic survey. Journal of Applied Psychology, 60(2), 159–170. 10.1037/h0076546

[bjep70069-bib-0022] Henrich, J. , & Mathukrishna, M. (2021). The origins and psychology of human cooperation. Annual Review of Psychology, 72, 207–240. 10.1146/annurev-psych-081920-042106 33006924

[bjep70069-bib-0023] Hertel, G. , Deters, C. , & Konradt, U. (2003). Motivation gains in computer‐supported groups. Journal of Applied Social Psychology, 33(10), 2080–2105. 10.1111/j.1559-1816.2003.tb01876.x

[bjep70069-bib-0024] Hertel, G. , Kerr, N. L. , & Messé, L. A. (2000). Motivation gains in performance groups: Paradigmatic and theoretical developments on the Kohler effect. Journal of Personality and Social Psychology, 79(4), 580–601. 10.1037//0022-3514.79.4.580 11045740

[bjep70069-bib-0025] Hertel, G. , Nohe, C. , Wessolowski, K. , Meltz, O. , Pape, J. , Fink, J. , & Hüffmeier, J. (2018). Effort gains in occupational teams – The effects of social competition and social indispensability. Frontiers in Psychology, 9, 769. 10.3389/fpsyg.2018.00769 29872412 PMC5972297

[bjep70069-bib-0026] Hüffmeier, J. , Hertel, G. , Torka, A.‐K. , Nohe, C. , & Krumm, S. (2022). In field settings group members (often) show effort gains instead of social loafing. European Review of Social Psychology, 33(1), 131–170. 10.1080/10463283.2021.1959125

[bjep70069-bib-0027] Inzlicht, M. , Werner, K. M. , Briskin, J. L. , & Roberts, B. (2021). Integrating models of self‐regulation. Annual Review of Psychology, 71, 319–345. 10.1146/annurev-psych-061020-105721 33017559

[bjep70069-bib-0028] Johnson, D. W. , & Johnson, R. T. (2002). Social interdependence theory and university instruction: Theory into practice. Swiss Journal of Psychology, 61(3), 119–129. 10.1024/1421-0185.61.3.119

[bjep70069-bib-0029] Johnson, D. W. , & Johnson, R. T. (2015). Cooperation and competition. In J. D. Wright (Ed.), International encyclopedia of the social & behavioral sciences (2nd ed., pp. 856–861). Elsevier. 10.1016/B978-0-08-097086-8.24051-8

[bjep70069-bib-0030] Johnson, D. W. , Johnson, R. T. , & Smith, K. (2007). The state of cooperative learning in postsecondary and professional settings. Educational Psychology Review, 19, 15–29. 10.1007/s10648-006-9038-8

[bjep70069-bib-0031] Kelley, H. H. , Holmes, J. G. , Kerr, N. L. , Reis, H. T. , Rusbult, C. E. , & van Lange, P. A. M. (2003). An atlas of interpersonal situations. Cambridge University Press. 10.1017/CBO9780511499845

[bjep70069-bib-0032] Kim, K. R. , & Seo, E. H. (2015). The relationship between procrastination and academic performance: A meta‐analysis. Personality and Individual Differences, 82, 26–33. 10.1016/j.paid.2015.02.038

[bjep70069-bib-0033] Klingsieck, K. B. , & Fries, S. (2012). Allgemeine prokrastination: Entwicklung und validierung einer deutschsprachigen kurzskala der General Procrastination Scale (Lay, 1986). [General procrastination: Development and validation of the German short scale of the General Procrastination Scale (Lay, 1986)]. Diagnostica, 58(4), 182–193. 10.1026/0012-1924/a000060

[bjep70069-bib-0034] Klingsieck, K. B. , Grund, A. , Schmid, S. , & Fries, S. (2013). Why students procrastinate: A qualitative approach. Journal of College Student Development, 54(4), 397–412. 10.1353/csd.2013.0060

[bjep70069-bib-0035] Koppenborg, M. , & Klingsieck, K. B. (2022). Group work and student procrastination. Learning and Individual Differences, 94, 102117. 10.1016/j.lindif.2022.102117

[bjep70069-bib-0036] Koppenborg, M. , Klingsieck, K. B. , & Hüffmeier, J. (2024). Conjunctive and additive group work reduce academic procrastination: Insights from a vignette study. Current Psychology, 43(2), 997–1010. 10.1007/s12144-023-04294-w

[bjep70069-bib-0037] Krohne, H. W. , Egloff, B. , Kohlmann, C.‐W. , & Tausch, A. (1996). Untersuchung mit einer deutschen version der “positive and negative affect schedule” (PANAS) [Investigation with the German version of the ‘positive and negative affect schedule’ (PANAS)]. Diagnostica, 42(2), 139–156.

[bjep70069-bib-0038] Lewin, K. (1951). Field theory in social science. McGraw‐Hill.

[bjep70069-bib-0039] Michinov, N. , Brunot, S. , Le Bohec, O. , Juhel, J. , & Delaval, M. (2011). Procrastination, participation, and performance in an online learning environment. Computers and Education, 56, 243–252. 10.1016/j.compedu.2010.07.025

[bjep70069-bib-0040] Milgram, N. A. , Dangour, W. , & Raviv, A. (1992). Situational and personal determinants of academic procrastination. The Journal of General Psychology, 119(2), 123–133. 10.1080/00221309.1992.9921166

[bjep70069-bib-0041] Morris, P. E. , & Fritz, C. O. (2015). Conscientiousness and procrastination predict academic coursework marks rather than examination performance. Learning and Individual Differences, 39, 193–198. 10.1016/j.lindif.2015.03.007

[bjep70069-bib-0042] Nordby, K. , Klingsieck, K. B. , & Svartdal, F. (2017). Do procrastination‐friendly environments make students delay unnecessarily? Social Psychology of Education, 20(3), 491–512. 10.1007/s11218-017-9386-x

[bjep70069-bib-0043] Oflazian, J. S. , & Borders, A. (2023). Does rumination mediate the unique effects of shame and guilt on procrastination? Journal of Rational‐Emotive & Cognitive‐Behavior Therapy, 41, 237–246. 10.1007/s10942-022-00466-y 35847054 PMC9274181

[bjep70069-bib-0044] Patzelt, J. , & Opitz, I. (2014). Deutsche Version des Academic Procrastination State Inventory (APSI‐d) [German Version of the Academic Procrastination State Inventory (APSI‐d)]. Gesis. 10.6102/zis139

[bjep70069-bib-0045] Penner, L. A. , Dovidio, J. F. , Piliavin, J. A. , & Schroeder, D. A. (2005). Prosocial behavior: Multilevel perspectives. Annual Reviews of Psychology, 56, 365–392. 10.1146/annurev.psych.56.091103.070141 15709940

[bjep70069-bib-0046] Priniski, S. J. , Rosenzweig, E. Q. , Canning, E. A. , Hecht, C. A. , Tibbetts, Y. , Hyde, J. S. , & Harackiewicz, J. M. (2019). The benefits of combining value for the self and others in utility‐value interventions. Journal of Educational Psychology, 111(8), 1478–1497. 10.1037/edu0000343 31772414 PMC6879189

[bjep70069-bib-0047] Pychyl, T. A. , Morin, R. W. , & Salmon, B. R. (2000). Procrastination and the planning fallacy: An examination of the study habits of university students. Journal of Social Behavior and Personality, 15(5), 135–150.

[bjep70069-bib-0048] Rabin, L. A. , Fogel, J. , & Eskine, K. (2011). Academic procrastination in college students: The role of self‐reported executive functions. Journal of Clinical and Experimental Neuropsychology, 33(3), 344–357. 10.1080/13803395.2010.518597 21113838

[bjep70069-bib-0049] Rahimi, S. , Hall, N. C. , & Sticca, F. (2023). Understanding academic procrastination: A longitudinal analysis of procrastination and emotions in undergraduate and graduate students. Motivation and Emotion, 47, 554–574. 10.1007/s11031-023-10010-9

[bjep70069-bib-0050] Roseth, C. J. , Johnson, D. W. , & Johnson, R. T. (2008). Promoting early adolescents' achievement and peer relationships: The effects of cooperative, competitive, and individualistic goal structures. Psychological Bulletin, 134(2), 223–246. 10.1037/0033-2909.134.2.223 18298270

[bjep70069-bib-0051] Senécal, C. , Julien, E. , & Guay, F. (2003). Role conflict and academic procrastination: A self‐determination perspective. European Journal of Social Psychology, 33(1), 125–145. 10.1002/ejsp.144

[bjep70069-bib-0052] Seta, J. J. (1982). The impact of comparison processes on coactors' task performance. Journal of Personality and Social Psychology, 42(2), 281–291. 10.1037/0022-3514.42.2.281

[bjep70069-bib-0053] Sirois, F. M. , & Pychyl, T. (2013). Procrastination and the priority of short‐term mood regulation: Consequences for future self. Social and Personality Psychology Compass, 7(2), 115–127. 10.1111/spc3.12011

[bjep70069-bib-0054] Springer, L. , Stanne, M. E. , & Donovan, S. S. (1999). Effects of small‐group learning on undergraduates in science, mathematics, engineering, and technology: A meta‐analysis. Review of Educational Research, 69(1), 21–51. 10.3102/00346543069001021

[bjep70069-bib-0055] Steel, P. (2007). The nature of procrastination: A meta‐analytic and theoretical review of quintessential self‐regulatory failure. Psychological Bulletin, 133(1), 65–94. 10.1037/0033-2909.133.1.65 17201571

[bjep70069-bib-0056] Steiner, I. (1972). Group process and productivity. Academic Press.

[bjep70069-bib-0057] Svartdal, F. , Dahl, T. , Gamst‐Klaussen, T. , Koppenborg, M. , & Klingsieck, K. B. (2020). How study environments foster academic procrastination: Overview and recommendations. Frontiers in Psychology, 11, 540910. 10.3389/fpsyg.2020.540910 33224046 PMC7667251

[bjep70069-bib-0058] Svartdal, F. , Sæle, R. G. , Dahl, T. I. , Nemtcan, E. , & Gamst‐Klaussen, T. (2022). Study habits and procrastination: The role of academic self‐efficacy. Scandinavian Journal of Educational Research, 66(7), 1141–1160. 10.1080/00313831.2021.1959393

[bjep70069-bib-0059] Torka, A. , Mazei, J. , & Hüffmeier, J. (2021). Together, everyone achieves more – Or, less? An interdisciplinary meta‐analysis on effort gains and losses in teams. Psychological Bulletin, 147(5), 504–534. 10.1037/bul0000251 34292010

[bjep70069-bib-0060] Van Eerde, W. , & Klingsieck, K. B. (2018). Overcoming procrastination? A meta‐analysis of intervention studies. Educational Research Review, 25, 73–85. 10.1016/j.edurev.2018.09.002

[bjep70069-bib-0061] Wang, Y. (2021). Academic procrastination and test anxiety: A cross‐lagged panel analysis. Journal of Psychologists and Counsellors in Schools, 31(1), 122–129. 10.1017/jgc.2020.29

[bjep70069-bib-0062] Watson, D. , Clark, L. A. , & Tellegen, A. (1988). Development and validation of brief measures of positive and negative affect: The PANAS scales. Journal of Personality and Social Psychology, 54(6), 1063–1070. 10.1037//0022-3514.54.6.1063 3397865

[bjep70069-bib-0063] Watson, D. , & Tellegen, A. (1985). Toward a consensual structure of mood. Psychological Bulletin, 98(2), 219–235. 10.1037/0033-2909.98.2.219 3901060

[bjep70069-bib-0064] Weber, B. , & Hertel, G. (2007). Motivation gains of inferior group members: A meta‐analytical review. Journal of Personality and Social Psychology, 93(6), 973–993. 10.1037/0022-3514.93.6.973 18072849

[bjep70069-bib-0065] Wieland, L. , Hoppe, J. D. , Wolgast, A. , & Ebner‐Priemer, U. W. (2022). Task ambiguity and academic procrastination: An experience sampling approach. Learning and Instruction, 81, 101595. 10.1016/j.learninstruc.2022.101595

[bjep70069-bib-0066] Wiese, C. W. , Shuffler, M. L. , & Salas, E. (2015). Teamwork and team performance measurement. In J. D. Wright (Ed.), International encyclopedia of the social & behavioral sciences (2nd ed., pp. 96–103). Elsevier. 10.1016/B978-0-08-097086-8.22017-5

[bjep70069-bib-0067] Wiwatowska, E. , Prost, M. , Coll‐Martin, T. , & Lupiáñez, J. (2025). Is poor control over thoughts and emotions related to a higher tendency to delay tasks? The link between procrastination, emotional dysregulation and attentional control. British Journal of Psychology, 116, 807–830. 10.1111/bjop.12793 40296374 PMC12514333

[bjep70069-bib-0068] Yang, X. , Zhu, J. , & Hu, P. (2023). Perceived social support and procrastination in college students: A sequential mediation model of self‐compassion and negative emotions. Current Psychology, 42, 5521–5529. 10.1007/s12144-021-01920-3

[bjep70069-bib-0069] Yeager, D. S. , Henderson, M. D. , Paunesku, D. , Walton, M. , D'Mello, S. , Spitzer, B. J. , & Duckworth, A. L. (2014). Boring but important: A self‐transcendent purpose for learning fosters academic self‐regulation. Journal of Personality and Social Psychology, 107(4), 559–580. 10.1037/a0037637 25222648 PMC4643833

