# Supplementary material for: Is procrastination among students lower in group work? Evidence from a registered field experiment
Source: Br J Educ Psychol. 2026 Mar 10;96(3):1102–22. doi: 10.1111/bjep.70069 (PMC13440998; doi:10.1111/bjep.70069)
Supplement: Supplementary file 1 [file BJEP-96-1102-s001.docx]

**Electronic Supplement to:**

**Is Procrastination Among Students Lower in Group Work? Evidence From a Registered Field Experiment**

**Content**

1. Details on a-priori power analysis
2. Table E1. Description of excluded participants
3. Details on assessing errors in the experimental task (i.e., the bibliography)
4. Items to assess perceived indispensability
5. Details on control variables
6. Table E2. Descriptive statistics for all dependent variables across conditions and sampling periods.
7. Table E3. Test statistics for all comparisons between sampling periods (winter vs. summer) across conditions and dependent variables

**1. Details on a-priori power analysis**

Assumed effect sizes for procrastination (*d* = 0.88) and positive affect (*d* = 0.78) were based on a previous vignette study on the comparison of conjunctive group work and individual work (cf. *reference blinded*). Assumed effect size for performance (*d* = 0.6) was based on a previous field experiment on the comparison of interdependent group work and individual work (cf. *reference blinded*). We used the smallest effect size (*d* = 0.6 for performance) as the basis for our power analysis. We did not use the effect size of indispensability perceptions as the basis for our power analysis, because our study was primarily designed to find out whether the predicted main effects would be observed at all (and indispensability perceptions as a potential mediator are only a secondary focus of our study).

Table E1. Description of excluded participants

|  | Number of excluded participants | | | |  |
| --- | --- | --- | --- | --- | --- |
|  | Total | Male | Female | Diverse | No answer |
| **Total** | 1,317 | 319 | 979 | 12 | 7 |
| **Age** |  |  |  |  |  |
| Below 18 years | 3 |  |  |  |  |
| 18–21 years | 529 |  |  |  |  |
| 22–24 years | 403 |  |  |  |  |
| 25–27 years | 223 |  |  |  |  |
| 28 years and above | 154 |  |  |  |  |
| No answer | 5 |  |  |  |  |
| **Programs** |  |  |  |  |  |
| Bachelor’s | 941 |  |  |  |  |
| Master’s | 331 |  |  |  |  |
| Other | 26 |  |  |  |  |
| No answer | 19 |  |  |  |  |
| **Year of study** |  |  |  |  |  |
| First | 419 |  |  |  |  |
| Second | 392 |  |  |  |  |
| Third | 242 |  |  |  |  |
| Above third | 230 |  |  |  |  |
| No answer | 34 |  |  |  |  |

**3. Details on assessing errors in the experimental task (i.e., the bibliography)**

For an entry in the bibliography to count as correct, it had to exactly accord with APA guidelines. Thus, a single deviation from the guidelines in one entry resulted in this entry to count as incorrect. The most typical deviations of our participants included (1) omission or incorrect usage of punctuation characters, for example, full points, commas or space characters, (2) incorrect usage of capitalisation in article titles and journal titles, (3) incorrect formatting, for example, italics and indents, and (4) incorrect spelling of author’s names.

**4. Items to assess perceived indispensability**

1. “How dispensable or indispensable is your contribution for the group result?” (cf. Hertel et al., 2018; *reference blinded*),
2. “How unimportant or important is your contribution for the group result?” (cf. Hertel et al., 2018; *reference blinded*),
3. “How important was it to you not to let your partners down when working on the task?”; cf. Hertel et al., 2008)

Items were rated on a five5-point scale ranging from 1 (*completely dispensable, very unimportant*) to 5 (*completely indispensable, very important*).

**References**

Hertel, G., Nohe, C., Wessolowski, K., Meltz, O., Pape, J., Fink, J., & Hüffmeier, J. (2018). Effort gains in occupational teams - The effects of social competition and social indispensability. *Frontiers in Psychology*. <https://doi.org/10.3389/fpsyg.2018.00769>

**5. Details on control variables**

*Preference for team work*: During the screening, participants’ self-ratings were assessed with three items from the Beliefs About Groups Scale (BAG; Karau & Elsaid, 2009; e.g., „I prefer group work to individual work”) that were selected based on their factor loadings, translated into German and that were rated on a 5-point scale ranging from 1 (*strongly disagree*) to 5 (*strongly agree*).

*Social comparison:* In the two group work conditions, social comparison with the other group members concerning the assignment was assessed with one item (“How important would it be for you to be better or at least not worse than the other group members in this task?”, cf. Hertel et al., 2018) that is rated on a 5-point scale ranging from 1 (*very unimportant*) to 5 (*very important)*.

*Extrinsic motivation*: In all three conditions, extrinsic motivation to complete the assignment was assessed with three items that were inspired by and adapted from the Work Extrinsic and Intrinsic Motivation Scale (Tremblay et al., 2009; e.g., “I participate in this study because it allows me to earn money”), that were rated on a 5-point scale ranging from 1 (*strongly disagree*) to 5 (*strongly agree*).

**References**

Hertel, G., Nohe, C., Wessolowski, K., Meltz, O., Pape, J., Fink, J., & Hüffmeier, J. (2018). Effort gains in occupational teams - The effects of social competition and social indispensability. *Frontiers in Psychology*. <https://doi.org/10.3389/fpsyg.2018.00769>

Karau, S. J., & Elsaid, A. M. M. K. (2009). Individual differences in beliefs about groups. Group Dynamics: Theory, Research, and Practice, 13(1), 1–13. <https://doi.org/10.1037/a0013366>

Tremblay, M. A., Blanchard, C. M., Taylor, S., Pelletier, L. G., & Villeneuve, M. (2009). Work extrinsic and intrinsic motivation scale: Its value for organizational psychology research. *Canadian Journal of Behavioural Science/Revue canadienne des sciences du comportement, 41*(4), 213–226.

Table E1. Descriptive statistics for all dependent variables across conditions and sampling periods.

|  | Individual work | | | |  | Conjunctive group work | | | |  | Additive group work | | | |
| --- | --- | --- | --- | --- | --- | --- | --- | --- | --- | --- | --- | --- | --- | --- |
|  | Sampling period | | | |  | Sampling period | | | |  | Sampling period | | | |
|  | Winter | | Summer | |  | Winter | | Summer | |  | Winter | | Summer | |
| **Dependent variables** | *N* | *M (SD)* | *N* | *M (SD)* |  | *N* | *M (SD)* | *N* | *M (SD)* |  | *N* | *M (SD)* | *N* | *M (SD)* |
| Task procrastination | 27 | 3.31 (1.14) | 37 | 2.84 (1.04) |  | 55 | 2.69 (0.95) | 3 | 2.58 (0.52) |  | 45 | 3.12 (1.07) | 8 | 2.34 (0.65) |
| Task performance | 39 | 4.35 (5.49) | 42 | 5.76 (5.99) |  | 68 | 5.82 (6.40) | 3 | 13.00 (4.33) |  | 56 | 5.18 (5.70) | 10 | 7.25 (6.52) |
| Positive affect (prosp.) | 39 | 2.84 (0.73) | 42 | 3.12 (0.82) |  | 68 | 2.82 (0.77) | 3 | 2.90 (0.62) |  | 56 | 2.90 (0.61) | 10 | 2.99 (0.58) |
| Positive affect (retrosp.) | 27 | 2.53 (0.87) | 37 | 2.61 (0.93) |  | 55 | 2.69 (1.05) | 3 | 2.73 (0.67) |  | 45 | 2.73 (0.80) | 8 | 2.46 (0.77) |
| Negative affect (prosp.) | 39 | 1.50 (0.61) | 42 | 1.42 (0.61) |  | 67 | 1.63 (0.71) | 3 | 2.20 (0.61) |  | 55 | 1.71 (0.69) | 10 | 1.74 (0.73) |
| Negative affect (retrosp.) | 27 | 1.76 (0.81) | 37 | 1.49 (0.65) |  | 55 | 1.62 (0.77) | 3 | 1.80 (0.36) |  | 44 | 1.70 (0.74) | 8 | 1.36 (0.70) |

Notes. Prosp. = prospective; retrosp. = retrospective

Table E2. Test statistics for all comparisons between sampling periods (winter vs. summer) across conditions and dependent variables

|  | Individual work | | Conjunctive group work | | Additive group work | |
| --- | --- | --- | --- | --- | --- | --- |
| **Dependent variables** | *Test statistic* | *p* | *Test statistic* | *p* | *Test statistic* | *p* |
| Task procrastination | *F*(1, 62) = 2.94 | .045 | *F*(1, 56) = 0.04 | .85 | *F*(1, 51) = 3.90 | .025 |
| Task performance | *F*(1, 79) = 1.22 | .14 | *F*(1, 69) = 3.66 | .03 | *F*(1, 64) = 1.07 | .30 |
| Positive affect (prosp.) | *F*(1, 79) = 2.57 | .11 | *F*(1, 69) = 0.03 | .87 | *F*(1, 64) = 0.19 | .67 |
| Positive affect (retrosp.) | *F*(1, 62) = 0.13 | .72 | *F*(1, 56) = 0.01 | .94 | *F*(1, 51) = 0.86 | .36 |
| Negative affect (prosp.) | *F*(1, 79) = 0.28 | .60 | *F*(1, 68) = 1.85 | .18 | *F*(1, 63) = .01 | .92 |
| Negative affect (retrosp.) | *F*(1, 62) = 2.55 | .11 | *F*(1, 56) = 0.16 | .69 | *F*(1, 50) = 1.62 | .21 |

Notes. Prosp. = prospective; retrosp. = retrospective
